# Engineering Wettability Transitions on Laser-Textured Shark Skin-Inspired Surfaces via Chemical Post-Processing Techniques

**DOI:** 10.3390/mi15121442

**Published:** 2024-11-28

**Authors:** Elham Lori Zoudani, Nam-Trung Nguyen, Navid Kashaninejad

**Affiliations:** Queensland Micro- and Nanotechnology Centre, Griffith University, Nathan Campus, 170 Kessels Road, Brisbane, QLD 4111, Australia; elham.lorizoudani@griffithuni.edu.au

**Keywords:** surface wettability, wetting transition, laser machining, shark skin-inspired microstructures, chemical post-processing

## Abstract

Surface wettability, the interaction between a liquid droplet and the surface it contacts, plays a key role in influencing droplet behavior and flow dynamics. There is a growing interest in designing surfaces with tailored wetting properties across diverse applications. Advanced fabrication techniques that create surfaces with unique wettability offer significant innovation potential. This study investigates the wettability transition of laser-textured anisotropic surfaces featuring shark skin-inspired microstructures using four post-processing methods: spray coating, isopropyl alcohol (IPA) treatment, silicone oil treatment, and silanization. The impact of each method on surface wettability was assessed through water contact angle measurements, scanning electron microscopy (SEM), and laser scanning microscopy. The results show a transition from superhydrophilic behavior on untreated laser-textured surfaces to various (super)hydrophobic states following surface treatment. Chemical treatments produced different levels of hydrophobicity and anisotropy, with silanization achieving the highest hydrophobicity and long-term stability, persisting for one year post-treatment. This enhancement is attributed to the low surface energy and chemical properties of silane compounds, which reduce surface tension and increase water repellence. In conclusion, this study demonstrates that post-processing techniques can effectively tailor surface wettability, enabling a wide range of wetting properties with significant implications for practical applications.

## 1. Introduction

Creating surfaces with varying wettability is an intriguing concept that warrants increased attention from the research community and the industry. Surfaces with distinct wetting properties offer diverse applications [1,2]. Superhydrophobic and hydrophobic surfaces (with water contact angles ranging from 90 degrees to above 150 degrees) are ideal for self-cleaning [3,4] and anti-biofouling [5,6]. Superhydrophilic surfaces are particularly suited for promoting unidirectional fluid flow, specifically in microfluidic applications [7,8]. Surface wetting is considered isotropic when the contact angle is identical in every direction. Conversely, anisotropic wetting is characterized by directional variations in the contact angle resulting from asymmetrical micro/nanostructures or specific chemical compositions [9].

Wettability can be regulated through the introduction of specific surface morphologies or chemical modifications. Achieving the desired wettability requires aligning the appropriate surface topology with suitable chemical characteristics [10,11]. Various fabrication techniques—including photolithography [12], micromilling [13], molding, and laser machining [14]—have been employed to generate these structures [15,16]. Additionally, numerous strategies have been explored for chemically altering surfaces to enhance or modify their wettability [17,18,19].

Among these methods, laser machining stands out as a fast, precise, versatile, and contactless approach. Notably, laser machining influences surface wettability by modifying both the physical and chemical properties of the material. When applied to metals, laser machining typically induces a superhydrophilic state due to the formation of metal oxides during the laser ablation process [20,21]. The stability of this superhydrophilic property depends heavily on environmental conditions. If the laser-textured surface is stored in controlled atmospheres such as O_2_, N_2_, or CO_2_, its hydrophilicity can be maintained [11]. However, exposure to ambient air leads to a gradual transition from superhydrophilicity to hydrophobicity, driven by environmental factors. Chemical modifications can further accelerate this wetting transition, transforming laser-textured surfaces from superhydrophilic to hydrophobic or even superhydrophobic states. Researchers have explored modifying laser-textured substrates to achieve (super)hydrophobic surfaces by employing various chemical post-treatments [22,23,24].

Nature is an abundant source of wetting surfaces with unique surface features, with great examples being plant leaves and the skin patterns of various organisms [25,26,27]. Drawing inspiration from nature, many researchers have explored new wetting modes by mimicking biological designs [28,29,30]. For instance, the unique pattern of shark skin has been studied for applications such as drag reduction [31] and antifouling [32]. In this study, we adopted a shark skin-inspired pattern to fabricate surfaces with microstructures tailored for specific wetting properties.

Using laser machining, a one-step method, we created a series of microriblet structures on silicon substrates, mimicking shark skin. The fabricated superhydrophilic structures were subjected to four chemical post-processing techniques: spray coating, IPA treatment, silicone oil treatment, and silanization. The dimensions of the microstructures ranged from 480 µm × 50 µm to 120 µm × 50 µm.

Scanning electron microscopy (SEM) and laser microscopy were used to examine the surface’s morphology. The chemical composition of the surfaces was also evaluated post-laser machining and after chemical treatments. Water contact angle measurements were taken at each stage to quantitatively determine how these processes influenced surface wettability.

This study highlights the potential of creating surfaces with a specific wettability. The versatility of the proposed laser fabrication and chemical post-processing techniques is a key advantage in developing innovative wetting surfaces.

## 2. Materials and Methods

### 2.1. Materials

The materials that have been used for this study are as follows: 4-inch silicon wafers with crystal orientation <100>, a commercial spray solution, silicone oil (viscosity 5 cSt (25 °C), SIGMA-ALDRICH, Co., St. Louis, MO, USA), isopropyl alcohol (IPA), ethanol, acetone, and Triethoxy (1H,1H,2H,2H-perfluoro-1-octyl) silane 97% (SIGMA-ALDRICH, Co., St. Louis, MO, USA).

### 2.2. Fabrication Process

#### 2.2.1. Femtosecond Laser Machining

**Figure 1:**
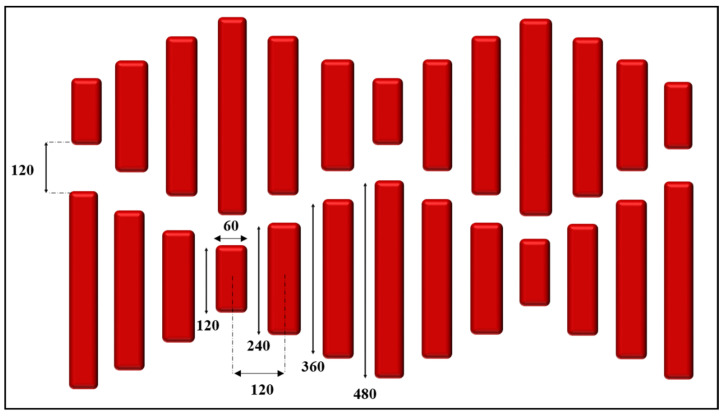
A schematic illustration of the pattern and dimensions of the shark skin design. The dimensions are given in micrometers.

A shark skin pattern containing a set of rectangular features with dimensions of 480 µm × 60 µm, 360 µm × 60 µm, 240 µm × 60 µm, and 120 µm × 60 µm (as shown in Figure 1) was fabricated using a femtosecond laser machine (A-Series, Oxford Lasers, Oxfordshire, UK). These dimensions were selected as the optimal values for maintaining the integrity of the structures. The initial step involved designing a 2D array of varying-sized rectangles, mimicking the shark skin pattern, using CAD/CAM software (AlphaCAM, 2021.0, HEXAGON, Tuscaloosa, AL, USA). The generated G-code was subsequently imported into Cimita laser micromachining software for laser processing.

Four sets of identical shark skin designs were fabricated for separate experimental conditions, each having the same exact dimensions and a scanning line thickness of 0.008 mm. The laser parameters were set to a power of 3 W and a speed of 4 mm/s. The parameters were selected as the optimal values for laser machining the structures.

#### 2.2.2. Post-Processing Procedure

**Figure 2:**
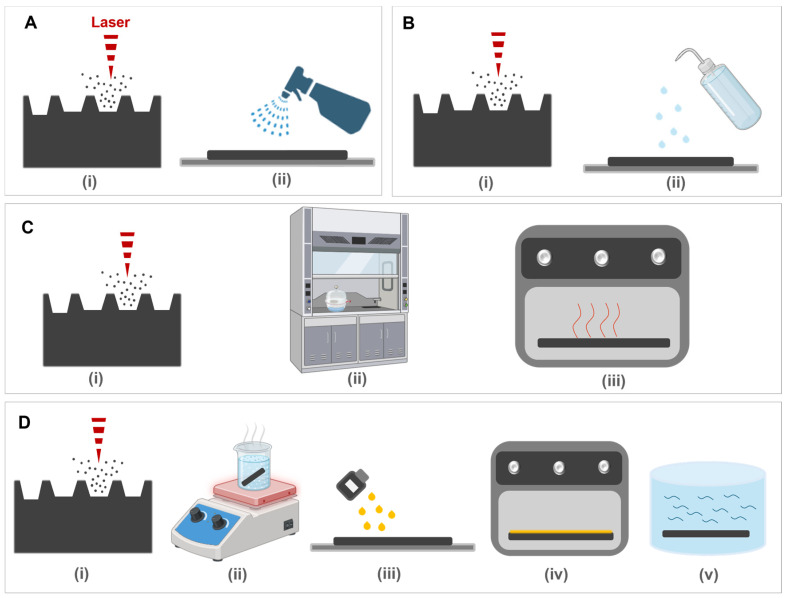
A schematic of the post-treatment experiments: (**A**) spray coating; (**B**) IPA coating; (**C**) silanization; (**D**) silicone oil treatment. Created in https://BioRender.com.

Four post-processing techniques—spray coating, IPA treatment, silicone oil treatment, and silanization—were employed in this study. The schematic representation of the processing steps for each method is illustrated in Figure 2.

i. Spray Coating and IPA treatment

The first two methods, IPA coating and the application of a commercial spray solution, were straightforward. In both cases, the respective solutions were applied directly onto the laser-machined patterns, as shown in Figure 2A,B. Following the application, the samples were left at room temperature under ambient conditions for several days to allow for natural drying and adhesion.

ii. Silanization

For the silanization process, the sample was treated with the ethanol–silane solution (Triethoxy (1H,1H,2H,2H-perfluoro-1-octyl) silane) and kept in the desiccator overnight. Afterwards, the sample was heated on a hot plate until the liquid evaporated. All of these steps involving silane were carried out under a fume hood. The samples were then placed in an oven at 100 °C for 2 h.

iii. Silicone Oil Treatment

The third method was adapted from [33] the work of who developed a silicone oil heat treatment process to convert laser-machined superhydrophilic patterns into superhydrophobic surfaces [33]. Figure 2D schematically demonstrates the steps of the experiment. In this technique, the sample was first immersed in boiling water for 10 min, then air-dried. Subsequently, the structure’s surface was coated with silicone oil and placed in an oven at 200 °C for 10 min. Finally, the sample underwent ultrasonic cleaning using a solution of acetone, IPA, and deionized (DI) water to remove excess oil.

### 2.3. Characterization Tests

The surface morphology of the laser-machined silicon wafer was analyzed using a scanning electron microscope (SEM) (Apreo 2S, Thermo Scientific, Waltham, MA, USA) and a 3D laser scanning microscope (LEXT 5100, Olympus, Tokyo, Japan). Chemical composition analysis was performed using the SEM’s energy-dispersive spectroscopy (EDS) function. The surface chemical composition was examined both after laser micromachining and post-processing treatment. Water contact angle (CA) measurements were conducted using an optical tensiometer (Theta Flex, Biolin Scientific, Espoo, Finland) with a 5 µL sessile droplet, both immediately after laser machining and following post-treatment, to assess the differences in surface wettability between the two conditions.

## 3. Results and Discussion

For the fabrication of shark skin patterns on the silicon surface, the same design was laser-machined onto four separate silicon wafers for subsequent post-processing experiments. All samples underwent post-treatment one day after laser texturing. The selected chemical treatments were designed to transition the surface properties from super-wettable to non-wettable states. The duration of this transition varied with treatments. The wetting transition occurred over 30 days for the IPA-treated sample and 3 weeks for the spray-coated sample, whereas surfaces treated with silicone oil or through silanization became hydrophobic immediately after the treatment. Characterization tests were performed for all samples following laser processing and chemical post-treatment. The results of surface analysis, focusing on morphological, chemical, and wettability properties, are presented in the subsequent sections. It should be noted that characterization tests were performed for each case immediately after the hydrophobic state was achieved.

### 3.1. Surface Morphology

**Figure 3:**
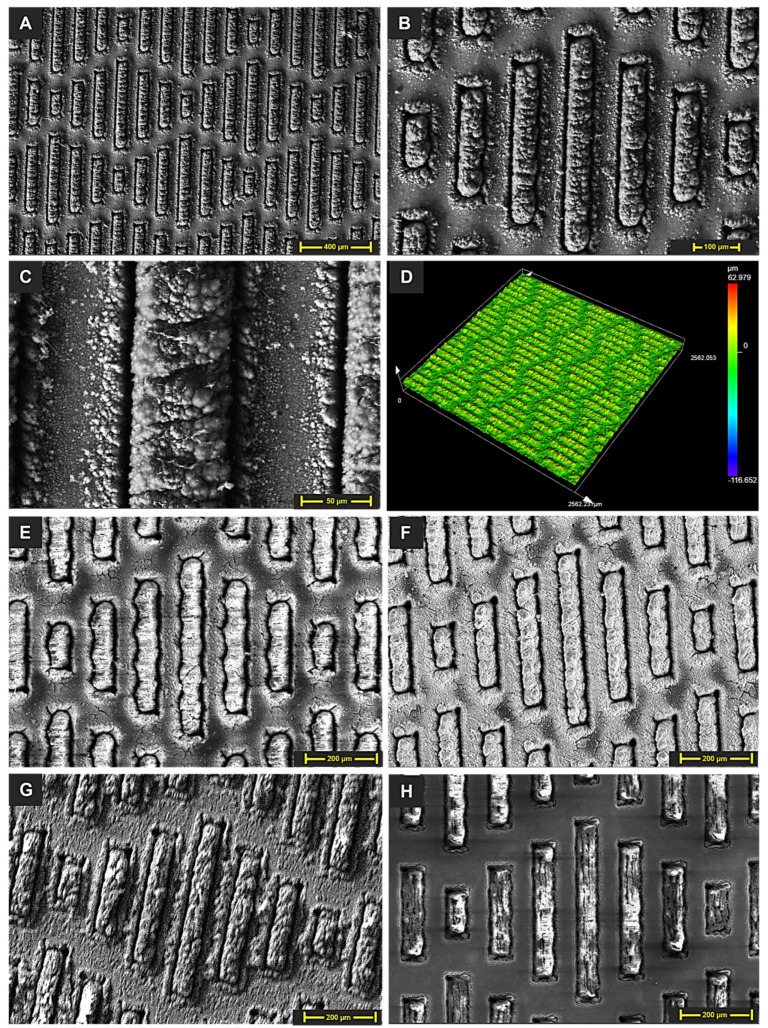
(**A**–**C**) SEM images of the fresh laser-textured shark skin design pattern. (**D**) A laser microscopy image of the fresh laser-textured sample. SEM images of the (**E**) spray-coated, (**F**) IPA-treated, (**G**) silanized, and (**H**) silicone oil-treated sample.

The surface topography of all samples, from fresh laser-textured structures to chemically modified ones, is illustrated in Figure 3. Figure 3A–D show the SEM and laser microscopy images of fresh laser-machined shark skin patterns. A closer look at the laser-machined pattern (Figure 3C) reveals visible micro- and nanostructures on the surface. During the laser ablation process, the increase in temperature caused the silicon material to melt, splash out, and solidify on the surface [21]. These micro/nanostructures on the laser-processed samples are likely responsible for the emergence of super-wettable behavior post-machining. The overall morphology of the fabricated pattern is clearly visible in these images. SEM images of the chemically modified structures (Figure 3E–H) demonstrate no significant change in surface topography between the freshly laser-machined and chemically treated samples.

### 3.2. Chemical Composition

**Figure 4:**
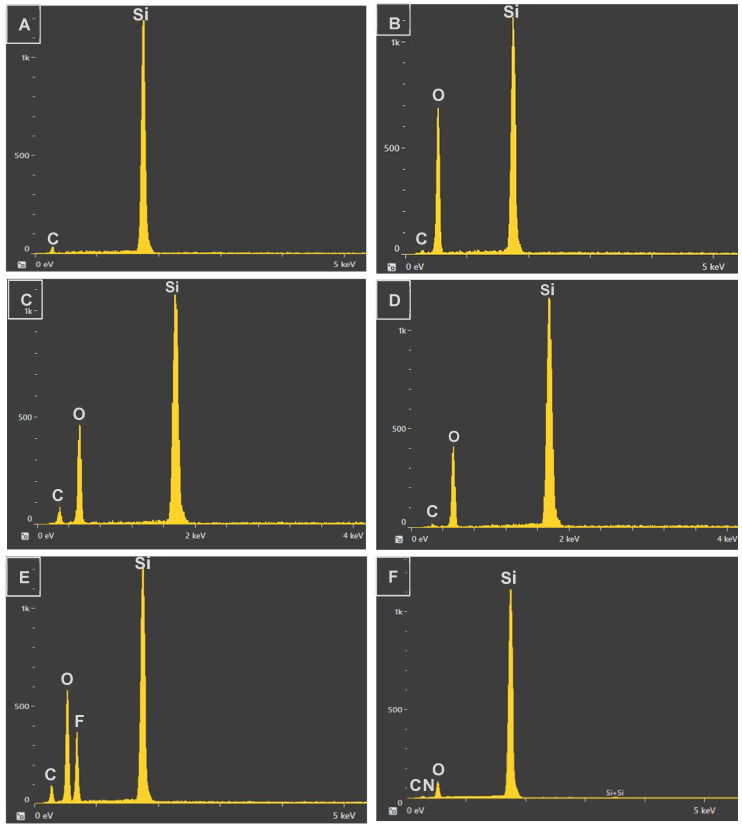
Chemical composition analysis: (**A**) pristine silicon wafer. (**B**) Fresh laser-machined sample. (**C**) Spray-coated sample. (**D**) IPA-treated sample. (**E**) Silanized sample. (**F**) Silicone oil-treated sample.

Figure 4 presents the chemical composition reports for all samples, including the pristine silicon substrate, the laser-machined shark skin micropatterns, and the samples following post-processing treatments. As discussed previously, laser processing can physically and chemically alter the surface properties of metals. The high-energy laser ablation process generates heat during machining, which reacts with the metal surface and leads to the formation of metal oxides in the treated areas. Figure 4A,B illustrate the chemical composition before and after laser texturing of the silicon substrate, confirming the presence of oxygen as a result of laser machining. Figure 4C–F display the elemental composition of the samples after chemical post-processing treatments, highlighting the changes induced by each method.

### 3.3. Water Contact Angle Measurement

As part of the characterization tests, the water contact angle (WCA) was measured for all surfaces using an optical tensiometer with 5 µL water droplets. These measurements were performed immediately after the wettability transition for each case. The tested surfaces included a pristine silicon wafer, a fresh laser-machined shark skin microstructure, and chemically modified samples (spray-coated, IPA-treated, salinized, and silicone oil-treated). Figure 6A presents the measured contact angle values for each case.

**Figure 5:**
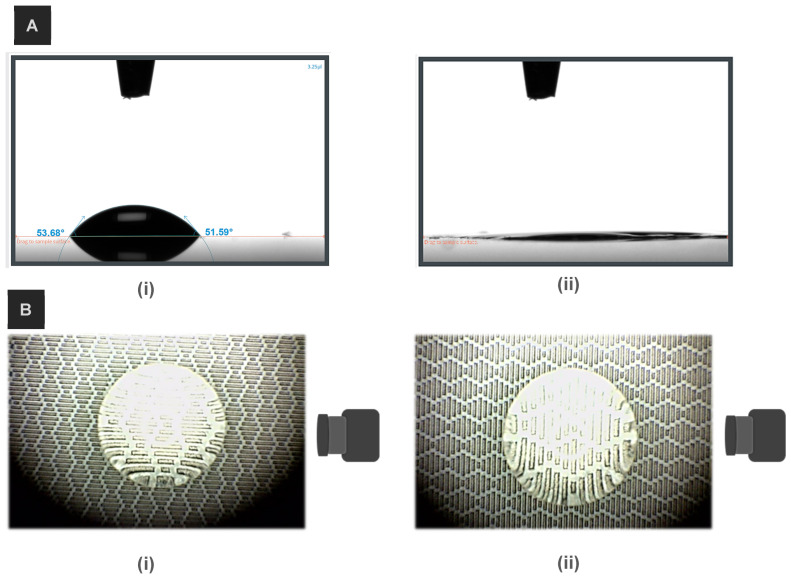
(**A**) Water contact angle on (**i**) pristine silicon wafer and (**ii**) freshly laser-machined shark skin pattern. (**B**) Top-view images of droplet contact angle captured from two perspectives: (**i**) parallel to ribs; (**ii**) perpendicular to ribs.

Figure 5A(i) shows that the bare silicon wafer exhibits hydrophilic behavior with an average contact angle of 52° ± 1. Laser machining transformed this hydrophilic surface into a superhydrophilic one. On the laser-machined surface, the water droplet could not maintain its spherical shape and completely spread out, resulting in a contact angle approaching zero, confirming the superhydrophilicity with wicking properties (Figure 5A(ii)).

The interaction between the droplet and the surface at each stage is governed by the chemistry of the top layer, which influences the wetting behavior [11]. The oxide layer generated by laser machining introduces polar functional groups, increasing the surface’s affinity for water and causing the water to spread upon contact [11]. However, post-processing treatment introduces a chemical layer that suppresses these polar functional groups, altering the surface’s interaction with water molecules and resulting in a transition to hydrophobicity.

The droplet state observed on these surfaces is primarily a result of a synergistic effect of their unique surface morphology (shark skin pattern) and chemical properties. According to the Cassie–Baxter model, the presence of micro/nanostructures on the surface creates air pockets beneath a droplet, positioning it on top of these air traps [34]. Additionally, the low-surface-energy chemical layer introduced on these structures further enhances water repellency by reducing water–surface adhesion and limiting droplet spread.

Surface wettability transitions, from superhydrophilicity to hydrophobicity or superhydrophobicity, were confirmed by examining the behavior of water droplets on the modified surfaces. To assess the degree of anisotropy in the shark skin microstructures, contact angle measurements were conducted in two different orientations. In one case, the camera was parallel to the ribs of the rectangular pattern, while in the other, the camera was positioned perpendicular to the rectangles. Figure 5B schematically illustrates these two orientations.

The measured contact angles for the chemically modified samples in the parallel orientation were 151° ± 1, 149° ± 2, 156° ± 2, and 144° ± 1 for the spray-coated, IPA-treated, salinized, and silicone oil-treated surfaces, respectively. In the perpendicular orientation, the contact angles were 148° ± 1, 144° ± 3, 154° ± 1, and 137° ± 1 for the same treatments. These results indicate that the degree of hydrophobicity varies across the different post-processing methods, with the salinized sample exhibiting the highest level of water repellence among all cases.

Moreover, the differences in contact angles between the two orientations reflect the anisotropy of the microstructures. The unique design of the shark skin pattern dictates this anisotropy, resulting in varying contact angles depending on the direction. It can be deduced from the reported data that each post-processing method imparts a unique anisotropic wetting property to the surface.

The analyzed methods were evaluated from multiple perspectives, including complexity, the time required for wettability transitions, the durability of the treated microstructures, and the overall wettability performance. Note that each of these factors plays a crucial role in selecting the most appropriate technique.

The complexity of each treatment method was assessed based on factors such as the required equipment and processing duration. The only essential tools for IPA and spray-coating treatments were the liquid solution and a sample holder, as both processes involved straightforward application of a solution onto the laser-textured surface. In contrast, the silicone oil treatment required additional equipment and more time per step, thereby increasing experimental complexity. Similarly, the salinization process required using a fume hood due to the hazardous nature of silane, as well as personal protective equipment (PPE), further adding to procedural complexity.

A major challenge with surface treatment techniques is ensuring the long-term stability of hydrophobicity, as coatings on microstructures may degrade over time due to environmental factors such as temperature and humidity. To fully assess the stability of wetting surfaces under various conditions, it is essential to conduct comprehensive mechanical wear, corrosion, and abrasion tests. These evaluations are critical for understanding how the surfaces perform under real-world scenarios, such as prolonged exposure to mechanical stresses, environmental pollutants, or abrasive interactions that may occur during practical use.

While the present study focuses on evaluating the wetting properties of these surfaces over time under ambient conditions, future investigations will explore mechanical wear resistance in greater depth. This will include assessing the durability of the surface textures and chemical modifications under simulated operational conditions, such as repeated physical contact, sliding, or exposure to harsh chemicals. Understanding these factors is crucial for enhancing the practical applicability and long-term performance of superhydrophobic surfaces in various applications, including antifouling, self-cleaning, and fluid handling technologies.

**Figure 6:**
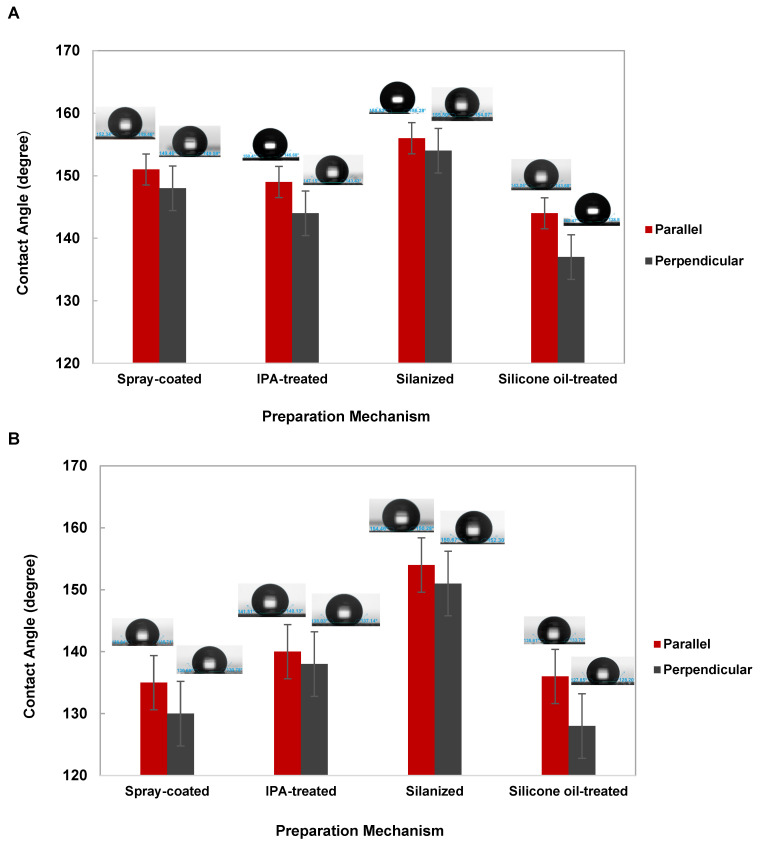
The measured water contact angle for four post-processed samples: spray-coated, IPA-treated, silanized, and silicone oil-treated. (**A**) Right after the wetting transition. (**B**) One year after the wetting transition. Different levels of hydrophobicity and anisotropy can be observed among the case studies. The contact angle measurements were conducted on five samples, and error bars show the standard deviation.

To evaluate hydrophobic durability, the treated surfaces were examined one year after post-processing. Contact angle measurement was conducted for all samples (Figure 6B). The contact angles in the parallel orientation were measured as 135° ± 1, 140° ± 1, 154° ± 2, and 136° ± 2 for spray-coated, IPA-treated, salinized, and silicone oil-treated samples, respectively. In the perpendicular orientation, the contact angles were 130° ± 1, 138° ± 1, 151° ± 1, and 128° ± 1 for the same treatments.

These findings highlight that silanization provides long-term hydrophobic stability. The durability of the hydrophobicity of these chemically post-processed samples is closely linked to the bonding strength of the chemical functional groups toward the laser-textured surface as well as environmental factors. The study controlled environmental conditions (all samples were stored in ambient air), ensuring consistent comparisons, and observed that variations in bonding strength between the introduced chemical layers and microstructures influenced the rate at which samples reverted to a more hydrophilic state.

A survey of the droplet state on chemically coated, laser-textured substrates can be conducted from the top (droplet) to the bottom (substrate). At the uppermost layer, the interaction occurs between the droplet and the coated layer. The droplet’s state on the surface is determined by molecular interactions between water molecules and the chemical coating. Each chemical layer used in the experiments introduces a unique functional group which in turn directly influences surface–droplet interactions and shapes the overall hydrophobicity

For example, fluorinated groups (e.g., C–F) exhibit higher superhydrophobicity compared to methyl groups (–CH_3_) from silicone oil, ester groups (–COOR), or hydroxyl groups (–OH) found in coatings from spray or isopropyl alcohol (IPA). Moving downward—from the droplet through the chemical coating to the laser-textured substrate—variations in functional groups result in distinct molecular bonding types (e.g., covalent, Van der Waals, hydrogen), directly affecting the stability of the coated chemical layer.

Among all tested cases, fluorinated functional groups demonstrate strong covalent bonding with the substrate. This high-affinity interaction forms a more stable layer compared to other functional groups.

A summary of the key parameters involved in the decision-making process for the post-processing methods is provided in Table 1, offering a comparative platform for evaluating these techniques from different aspects.

## 4. Conclusions

The present study highlights the importance of chemical modification techniques in controlling the wettability of laser-textured silicon surfaces with shark skin-inspired microstructures. Four post-processing methods—spray coating, IPA treatment, silicone oil treatment, and salinization—were applied, each demonstrating different transition times from a superhydrophilic to a hydrophobic or superhydrophobic state. Contact angle measurements revealed that the level of hydrophobicity and anisotropy varied across techniques, influenced by both the post-processing method and the surface geometry.

Among the methods, silanization achieved the highest hydrophobicity, with average contact angles of 156° and 154° for parallel and perpendicular orientations, respectively, but presented certain safety challenges. While IPA treatment and spray coating offered safer, environmentally friendly alternatives, they resulted in lower hydrophobicity. These findings underscore the need to balance performance, stability, and safety when selecting post-processing methods for tailored surface wettability.

## Figures and Tables

**Table 1:** Key parameters involved in post-processing experiments.

	Preparation Technique	Spray Coating	IPA Modification	Salinization	Silicone Oil Treatment
Criteria					
**Time required for wetting transition**		3 weeks	30 Days	After experiment	After experiment
**Measured water contact angle**		151° ± 1 (Parallel)148° ± 1 (Perpendicular)	149° ± 2 (Parallel)144° ± 3 (Perpendicular)	156° ± 2 (Parallel)154° ± 1 (Perpendicular)	144° ± 1 (Parallel)137° ± 1 (Perpendicular)
**Stability of the surface’s wettability**		Fourth rank in the category	Second rank in the category	First rank in the category	Third rank in the category
**Level of complexity of the experiment**		Low	Low	Highest	Comparatively high

## Data Availability

The essential data are contained within the article. The raw data are available on request from the corresponding authors.
